# PET/CT-negative malignant spine tumor with pathologic fracture: A case report of malignant solitary bone plasmacytoma

**DOI:** 10.1097/MD.0000000000013374

**Published:** 2018-12-14

**Authors:** Kang-Un Kim, Joon Hyuk Choi, Gun Woo Lee

**Affiliations:** a Department of Orthopaedic Surgery, Armed Forces Capital Hospital, Sungnam; b Department of Pathology, Yeungnam University Medical Center, Yeungnam University College of Medicine, Daegu; c Department of Orthopaedic Surgery, Spine Center, Yeungnam University Medical Center, Yeungnam University College of Medicine, Daegu, Republic of Korea.

**Keywords:** discrepancy, malignant, MRI, PET/CT, solitary plasmacytoma, spine tumor

## Abstract

**Rationale:**

We report on a patient with a positron emission tomography/computed tomography scans (PET/CT)-negative malignant spine tumor, which had even caused a pathologic fracture, and was eventually confirmed on surgical biopsy.

**Patient concerns:**

A 67-year-old man visited our emergency department with sudden onset of lower extremities paraplegia after slip down. On examination, gradually increasing paralysis was observed in both lower limbs.

**Diagnoses:**

Plain radiograph and CT showed an acute burst fracture at T12 with an osteolytic mass lesion within the vertebral body and pedicle, causing severe encroachment of the spinal canal. Magnetic resonance imaging (MRI) revealed a bulging posterior cortex of the T12 vertebral body, which suspected a pathologic fracture due to malignancy. However, PET/CT showed a benign burst fracture, which was confirmed by a senior radiologist.

**Intervention:**

We planned surgery for emergent decompression of the spinal cord, temporary stabilization, and tissue biopsy. The histologic evaluation confirmed the lesion to be a malignant solitary bone plasmacytoma (SBP). Seven days later, definite surgery in the form of pedicle screw fixation and posterolateral bone graft from T8 to L2 was performed. Four weeks after the definite surgery, the patient underwent radiation therapy for 2 months.

**Outcomes:**

Three weeks postoperatively, lower extremity motor function fully recovered, and ambulation with support was possible. One year postoperatively, spine MRI showed no evidence of local recurrence, and complete decompression of the spinal cord was achieved.

**Lessons:**

Spine surgeons should bear in mind that malignant spine tumors could be misinterpreted as benign on PET/CT.

## 1 Introduction

The use of ^18^fluorine-fluorodeoxyglucose positron emission tomography (^18^FDG PET) combined with computed tomography (CT) has been expanding gradually, especially for the purpose of non-invasive detection of malignancies and the confirmation of metastatic lesions for staging.^[1–4]^ However in rare instances, fluorodeoxyglucose (FDG) uptake can be misleading, resulting in the misinterpretation of some benign lesions as being malignant (false positive),^[4,5]^ and vice versa.^[5,6]^

Malignant spine tumors generally show increased uptake on PET/CT because of the hypermetabolic activity observed in a majority of malignant tumors. Moreover, the co-existence of pathologic fractures can enhance the detection of malignant lesions.^[1,7]^ Some studies have reported that a minority of malignancies can present as benign lesions on PET/CT, but a case of a malignant spine tumor with pathologic fracture misdiagnosed as a benign fracture has not yet been reported. We encountered a malignant spine tumor that appeared to be benign on PET/CT, despite having caused a pathologic fracture. Malignancy was finally confirmed by surgical biopsy. Our report highlights the fact that rare malignant tumors of the spine with pathologic fractures can also be misinterpreted as a benign fracture.

## 2 Case report

A 67-year-old man visited the emergency department of our hospital due to sudden paraplegia of both lower extremities caused immediately after slip down. On presentation to the department, both his lower limbs were observed to be almost paralyzed, with a motor grade of 2 or lower in both limbs. This paralysis showed a gradual aggravation. The patient had a history of renal cell carcinoma diagnosed 7 years prior, and achieved remission without any recurrence after total nephrectomy and adjuvant chemotherapy. He was also diagnosed with papillary thyroid carcinoma 5 years ago, and yet again achieved remission after surgical treatment. The patient signed an informed consent statement, and the study was approved by the Institutional Review Board of the Yeungnam University Medical Center.

**Figure 1:**
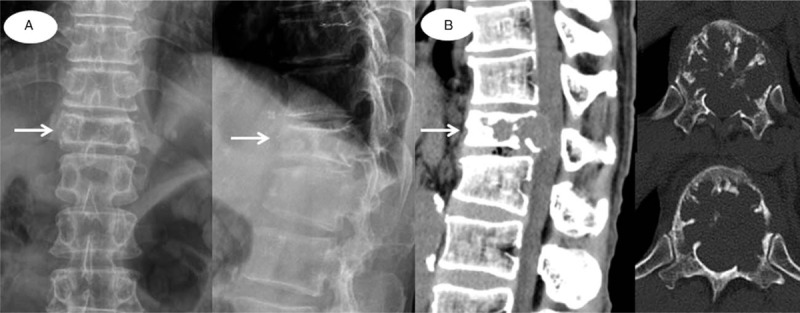
(A) Simple radiographs (anteroposterior and lateral) showed the decreased height of the T12 vertebral body. (B) CT images showed a burst fracture of the T12 vertebral body with an osteolytic lesion involving the vertebral body and pedicle, which is termed a “mini-brain appearance”. CT = computed tomography.

**Figure 2:**
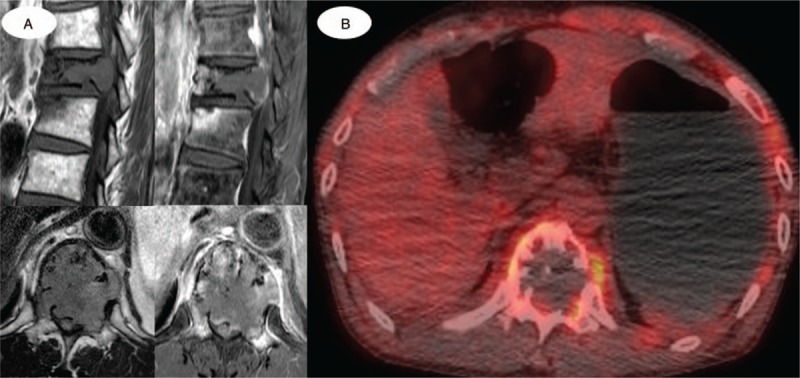
(A) Sagittal and axial MRI images showed the involvement of the entire T12 vertebra and severe canal encroachment, which was highly suspected to be caused by a malignant condition such as metastatic or primary cancer. (B) PET/CT demonstrated a benign condition with a maxSUV of 1.7. CT = computed tomography, PET = positron emission tomography.

**Figure 3:**
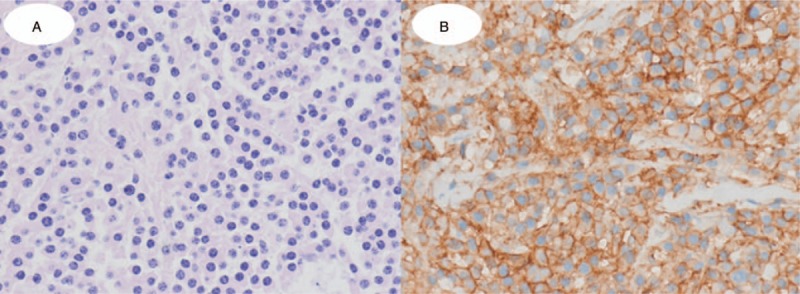
Histologic examination images. (A) The plasma cells are well differentiated, with eccentrically located nuclei and clock-face chromatin, and basophilic cytoplasm (H&E stain, ×400). (B) The plasma cells are positive for CD38 (Immunohistochemical stain, ×400).

The plain radiograph revealed the collapse of the T12 vertebral body. CT showed an acute burst fracture at the T12 vertebral body with an osteolytic mass-like lesion within the vertebral body and pedicle, causing severe encroachment of the spinal canal (Fig. 1). Magnetic resonance imaging (MRI) revealed a bulging posterior cortex, with an acute fracture of the vertebral body and severe compression of the spinal cord (Fig. 2). Considering his medical history and the MRI findings, a pathologic fracture with a tumorous condition was suspected. Therefore, the evaluation of the malignant potential and metastasis in the remote area was necessary. Hence, ^18^FDG PET/CT was performed, and ^18^FDG uptake at the T12 level was measured. A maximum standardized uptake value (maxSUV) of 1.7 was noted, with a central FDG defect on the vertebral body (Fig. 2); this was interpreted as a benign fracture by a senior radiologist. Additionally, there was no positive uptake in the other body parts on PET/CT. Although there was a discrepancy between the interpretation of the spine fracture lesion on MRI and PET/CT, the patient required emergent surgical treatment owing to acute paraplegia caused by spinal cord compression, and we thus planned an initial surgery for the main purpose of cord decompression, temporary stabilization, and tissue biopsy. The surgical intervention was as follows. First, we performed T11-12 posterior laminectomy for the spinal cord decompression, followed by T9-L2 posterior instrumentation without a bone graft. Finally, tissue biopsy was obtained at the T12 pedicle and vertebral body. Histological examination confirmed the presence of a malignant solitary bone plasmacytoma (SBP) (Fig. 3). Seven days later, a definite surgery with pedicle screw fixation and posterolateral bone graft from T8 to L2 without vertebral body corpectomy was performed.

### 2.1 Postoperative course

**Figure 4:**
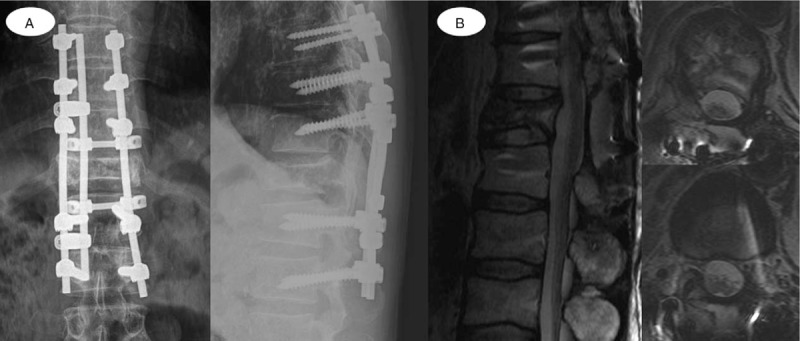
(A) One year postoperatively, simple radiographs (anteroposterior and lateral image) revealed that solid fusion was achieved, with no structural abnormality. (B) Spine MRI 1 year postoperatively showed complete decompression of the spinal cord and resolution of the malignant solitary bone plasmacytoma. MRI = magnetic resonance imaging.

Immediately after the initial surgery, the patient's lower extremity motor function showed gradual improvement. After the surgery, the patient wore a thoracolumbar rigid brace for 2 months, and underwent continuous gait rehabilitation. Three weeks postoperatively, the motor function in his lower extremities had recovered sufficiently, and ambulation was possible with the assistance of a cane. Four weeks after the definite surgery, the patient underwent radiation therapy for 2 months. One year postoperatively, spine radiographs showed that the surgical segment had healed fully, and spine MRI revealed complete decompression of the spinal cord with no evidence of local recurrence (Fig. 4).

## 3 Discussion

Our case highlights the fact that the ^18^FDG uptake observed on PET/CT is sometimes not malignancy-specific, and thus malignant lesions could sometimes be misinterpreted as being benign.^[1,4,7–9]^ Known factors contributing to false negative results on ^18^FDG PET imaging are the presence of low-grade tumor, tumor with low glycolytic activity, and small-sized tumor less than 1 cm in diameter.^[10,11]^ However, in our case, the size of the tumor was large enough to cause a pathologic fracture. Histological analysis of the tissue biopsy revealed malignant SBP. Malignant SBP is an extremely uncommon monoclonal disorder, and generally shows a positive uptake in most cases of ^18^FDG PET/CT.^[12]^ However, about 10% of SBP cases reported false-negative findings on ^18^FDG PET/CT.^[13–16]^

On ^18^FDG PET/CT images, the maxSUV difference in ^18^FDG uptake can distinguish between benign and malignant lesions in many cases.^[1,2,7,8,17]^ MRI is also a good tool to differentiate primary or metastatic malignant spine tumors from benign compression fractures. However, occasionally, this distinction is difficult.^[8]^ In patients with false negative ^18^FDG uptake, a discrepancy between MRI findings and PET uptake may be observed, such as in our case. Moreover, the false positive uptake of ^18^FDG could be seen in some cases of benign acute compression fractures.^[7,10,11]^ If such a discrepancy is present between MRI and PET/CT findings, the treatment plan, including surgery, should be based on the assumption that a malignant lesion might exist, and definitive treatment should be carried out after accurate diagnosis by histologic confirmation.

In summary, we report a case of false negative uptake on ^18^FDG PET/CT by a malignant neoplasm, which was confirmed as a malignant SBP in the T12 vertebra by histological examinations. Surgeons should always keep in mind that malignant spine lesions might be misinterpreted as being benign on ^18^FDG PET/CT.

## Author contributions

**Conceptualization:** Kang-Un Kim and Gun Woo Lee.

**Data curation:** Joon Hyuk Choi and Gun Woo Lee.

**Formal analysis:** Gun Woo Lee.

**Methodology:** Gun Woo Lee.

**Resources:** Gun Woo Lee

**Writing – original draft:** Kang-Un Kim, Joon Hyuk Choi, and Gun Woo Lee.
