# PET/CT-negative malignant spine tumor with pathologic fracture: A case report of malignant solitary bone plasmacytoma: Erratum

**DOI:** 10.1097/MD.0000000000014036

**Published:** 2018-12-28

In the article, “PET/CT-negative malignant spine tumor with pathologic fracture: A case report of malignant solitary bone plasmacytoma”,^[1]^ which appears in Volume 97, Issue 50 of *Medicine*, the authors would like to add the following sentence to the Acknowledgements, “This work was supported by the National Research Foundation of Korea (NRF) grant funded by the Korean Government and Ministry of Education (2017R1D1A1B03030530). The funder had no role in the study design, data collection and analysis, and study progression.”
